# Supplementary figures and images for: Detection of six soil-transmitted helminths in human stool by qPCR- a systematic workflow
Source: PLoS One. 2021 Sep 30;16(9):e0258039. doi: 10.1371/journal.pone.0258039 (PMC8483301; doi:10.1371/journal.pone.0258039)

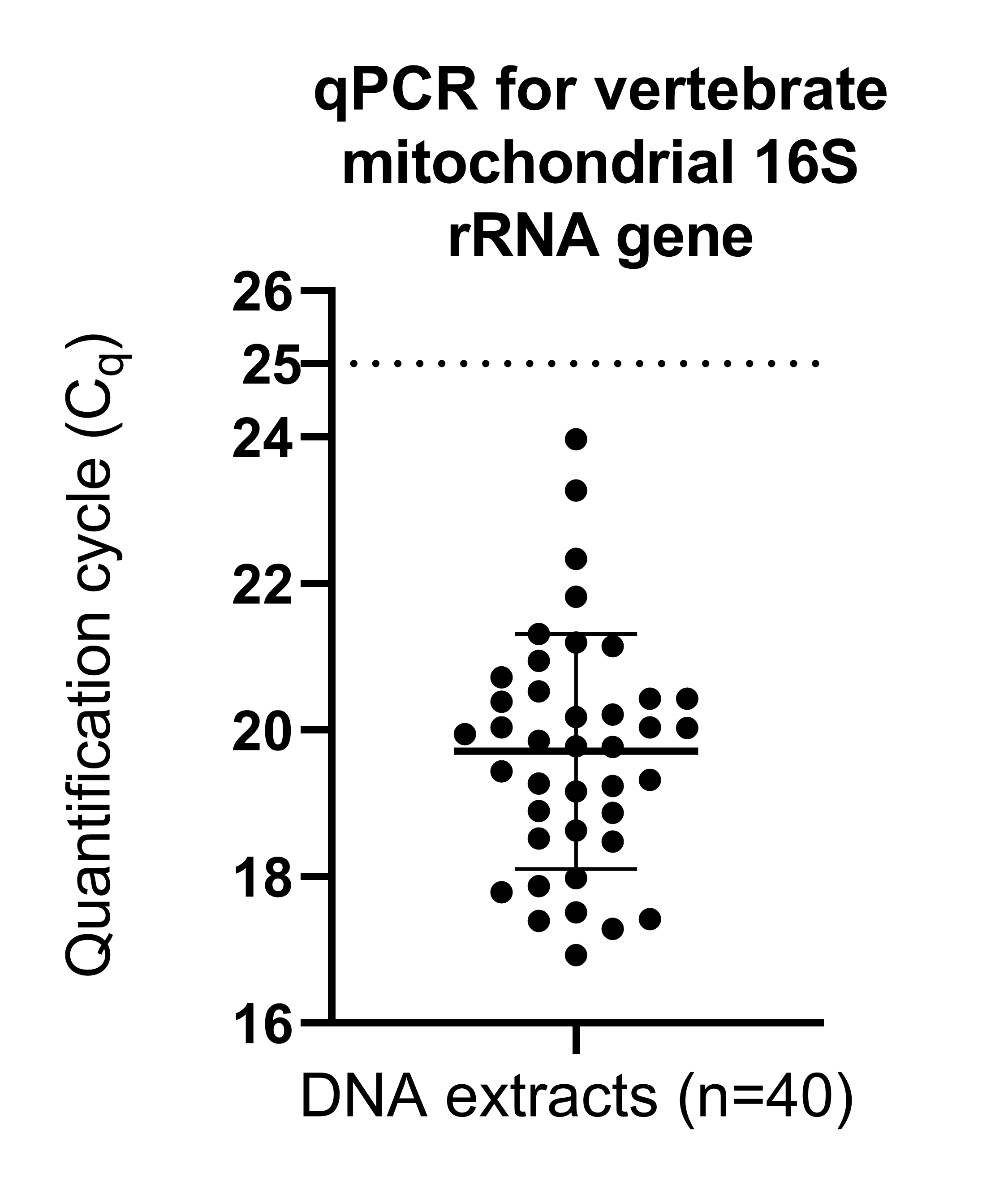

Supplement: S1 Fig — Stool DNA with Cqs over 25 (mean+3SD, dotted line) underwent repeat DNA extraction. Mean with standard deviation derived using duplicate qPCRs. (TIF) [file pone.0258039.s001.tif]
