# Supplementary material for: Detection of six soil-transmitted helminths in human stool by qPCR- a systematic workflow
Source: PLoS One. 2021 Sep 30;16(9):e0258039. doi: 10.1371/journal.pone.0258039 (PMC8483301; doi:10.1371/journal.pone.0258039)
Supplement: S1 Table — The FastPrep-24™ 5G (FP) and Mini-Beadbeater-24 (BB) were used in conjunction with the Isolate II Fecal Kit to detect S. stercoralis (Ss) larvae and A. lumbricoides (Al) extracted from human stool by qPCR. Mean Cq values and standard deviations from triplicate wells are shown. (PDF) [file pone.0258039.s002.pdf]

		1 cycle 40 s			2 cycles of 40 s			3 cycles of 40 s			4 cycles of 30 s		
Strongyloides stercoralis	FastPrep-24™ 5G	27.72	27.59	27.53	26.9	26.66	26.99	27.19	27.16	27.3	26.08	25.87	26.11
	Mini-Beadbeater-24	27.05	26.96	26.96	27.67	27.66	27.53	26.62	27.49	27.74	26.85	26.78	26.98
Ascaris lumbricoides	FastPrep-24™ 5G	32.62	32.66	32.97	32.42	32.1	31.43	32.54	32.01	31.68	32.31	32.26	32.56
	Mini-Beadbeater-24	32.18	32.34	32.35	32.69	32.9	32	33.7	32.9	33.26	32.36	32.88	32.76
