# Supplementary material for: Detection of six soil-transmitted helminths in human stool by qPCR- a systematic workflow
Source: PLoS One. 2021 Sep 30;16(9):e0258039. doi: 10.1371/journal.pone.0258039 (PMC8483301; doi:10.1371/journal.pone.0258039)
Supplement: S2 Table — (PDF) [file pone.0258039.s003.pdf]

S2 Table. The gBlock gene sequences used in this study.

	gBlock® gene	Sequence
**1**	Vertebrate mitochondrial 16S rRNA	ATTCTAGAGTCCATATCAACAATAGGGTTTACGACCTCGATGTTGGATCAGGAC<br>ATCCCGATGGTGCAGCCGCTATTAAAGGTTCGTTTGTTCAACGATTAAAGTCCTA<br>CGTGATCTGAGTTCAGACCGGAGTAATCCAGGTCGGTTTCTATCTACTTCA
**2**	*S. stercoralis*	TACGTAACTGGGAATGAAAATTGCAATTATTTTTCATGAACGAGGAATTCCAAG<br>TAAACGTAAGTCATTAGCTTACATTGATTACGTCCCTGCCCTTTGTACACACCGG<br>CCGTCGCTGCCCGGAACTGAGCAATATCCAGAGGCAGGAAGAGATGTAATAAA<br>TTTTTAATTTTTTTTATATTAAATCCTTCCAATCGCTGTTGTTTGAACCGGGCAAA<br>AGTCGTAACAAGGTTTTCGTAGGTGAACCTGCAGAAGGATCATCA
**3**	*T. trichiura*	ATGTTGGTGAATCGGAATAACTATGCTGATCGCACGGTCCAGTACCGGCGACGA<br>TGCTTTGAAACGACTTGCTCATCAACTTTCGATGGTACGCTACGTGCTTACCATG<br>GTGACAACGGTTAACGGAGAATCAGGGTTCGGCTCCGGAGAGGGAGCCTGAGA<br>AACGGCTACCACATCCAAGGAAGGCAGCAGGCACGCAAATTACCCACTCCCAG<br>AT
**4**	*A. lumbricoides*	TTCCGTAGGTGAACCTGCGGAAGGATCATTATCGAGCAGAAAAAAAAAAGTCT<br>CCGAACGTGCACATAAGTACTATTTGCGCGTATACGTGAGCCACATAGTAAATT<br>GCACACAAATGTGGTGATGTAATAGCAGTCGGCGGTTTCTTTTTTTTTGGCGGAC<br>AATTGCATGCGATTTGCTATGTGTTGAGGGAGAATAGGTGGCATGTTGGGCTTG<br>TTAGAAAGGCATGCCGCTAGCGCTTATTTTCCCGCTATTTCGTAACAACGGTGTC<br>CATTTTGGCGTCTACGCTTCACCGAGCTATCGCCTGGACCGTCGGTAGC
**5**	*A. duodenale*	TTGTCGAAGCCTTATGGTTCCTTTGATCCTGAGAAACCAACGTGCTAGTCTTCAC<br>GACTTTGTCGGGAAGGTTGGGAGTATCGCCCCCCGTTATAGCCCTACGTAAGGT<br>GTCTATGTGCAGCAAGAGTCGTTACTGGGTGACGGCAGTGATTGCTGTGCGAAG<br>TTCGCGTTTCGCTGAGCTTTAGACTTGATGAGCATTGCATGAATGCCGCCTTACT<br>GCTTGTG
**6**	*A. ceylanicum*	CGTGCTAGTCTTCAGGACTTTGTCGGGAAGGTTGGGAGTATCGCCCCCCGTTAC<br>AGCCCTACGTGAGGTGTCTATGTGCAGCAAGAGCCGTTCCTGGGTGGCGGCAGT<br>GATTGCTGTGCGAAGTTCGCGTTTCGCTGAGCTTTAGACTTGATGAGCATTGCAT<br>GAATGCCGCCTTACTGCTTGTGTTGGTGGTTGAGCA
**7**	*N. americanus*	AACGATAATACTACAGTGTAGCTTGTGGACAGTACTCTCACCGAGTATTGTCGA<br>ACACTGTTTGTCGAACGGTACTTGCTCTGTACTACGCATTGTATACGTGTTCAGC<br>AATTCCCGTTTAAGTGAAGAACACACGTGCAACATGTGCACGCTGTTATTCACT<br>ACGTTAGTTGCTAGTTTACTAACGTATGATAGCGGTGCATACTGTATGACATGA<br>ACATATCGTT
**8**	Equine herpesvirus type 4	GACGATGATGACACTAGCGACTTCGATGAAGCCAAGCTGGAGGAGGCACGCGA<br>AATGATCAAATATATGTCTATGGTTTCTGCCCTGGAAAAACAGGAAAAAAAGGC<br>AATGAAGAAAAACAAGGGGGTTGGACTTATTGCCAGCAACGTTTCAAAACTCG
