# Supplementary material for: Detection of six soil-transmitted helminths in human stool by qPCR- a systematic workflow
Source: PLoS One. 2021 Sep 30;16(9):e0258039. doi: 10.1371/journal.pone.0258039 (PMC8483301; doi:10.1371/journal.pone.0258039)
Supplement: S3 Table — (PDF) [file pone.0258039.s004.pdf]

**Mt 16S rRNA qPCR**	**STH1 quantification cycle**			**STH2 quantification cycle**			**Kato-Katz result**
C_q	*A. lumbricoides*	*S. stercoralis*	*T. trichiura*	*A. duodenale*	*A. ceylanicum*	*N. americanus*	Species (EPG)
23.3						34.19	Hookworm (36)
18.9							Negative
17.4						26.54	Hookworm (312)
18.9							Negative
21.0							Negative
19.3							*Trichuris* (144)
20.4							Hookworm (36)
16.9	36.77					34.75	*Ascaris* (300), hookworm (300), *Trichuris* (204)
18.5						26.47	Negative
20.0					31.27		Negative
21.3							Negative
20.2							Negative
18.6	29.7					29.38	*Ascaris* (624), hookworm (228)
19.2							Negative
24.0							Negative
21.0							Negative
17.3						28.59	Negative
19.4	29.61					27.88	Negative
17.9							*Trichuris* (48)
18.5							*Ascaris* (396), hookworm (600)
18.0							*Ascaris* (120), *Trichuris* (384)
19.9							*Trichuris* (48)
20.7							Negative
17.4					28.64		Hookworm (72)
20.2							Hookworm (96)
21.8							Negative
19.8							Negative
20.4					28.39	33.78	Hookworm (636)
19.8					31.44		Negative
17.5							Negative
17.8							Negative
21.2	36.22	35.09					*Ascaris* (108)
19.2					30.09		Negative
19.3	32.95						*Ascaris* (336)
20.0					22.68		Negative
20.4					31.42		Negative

**Mt 16S rRNA qPCR**	**STH1 quantification cycle**			**STH2 quantification cycle**			**Kato-Katz result**
C_q	*A. lumbricoides*	*S. stercoralis*	*T. trichiura*	*A. duodenale*	*A. ceylanicum*	*N. americanus*	Species (EPG)
20.5							*Trichuris* (192)
22.3							Negative
21.2	30.12						*Ascaris* (1224)
20.2	31.12						*Ascaris* (144)
