# Supplementary material for: Detection of six soil-transmitted helminths in human stool by qPCR- a systematic workflow
Source: PLoS One. 2021 Sep 30;16(9):e0258039. doi: 10.1371/journal.pone.0258039 (PMC8483301; doi:10.1371/journal.pone.0258039)
Supplement: Attachment — Submitted filename: Response to reviewers.docx [file pone.0258039.s005.docx]

# Reviewers’ comments with author response

# Methods

## Reviewer #1

line 88 - perhaps rephrase = low sensitivity in endemic populations with low intensity infections

Sentence amended to reflect reviewer’s suggestion (line 71):

“low sensitivity in endemic populations with low intensity infection”

Line 118-119 actually, the reason of preservations is to avoid DNA degradation, and deactivate nucleases found in faeces.
Faeces are full of microbes anyway - if the qPCR primers/probes are specific no need to worry about any overgrowth

Sentence amended to reflect reviewer’s suggestion as follows (line 103-104):

“Firstly, stool is collected and preserved to avoid DNA degradation and deactivate nucleases found in faeces.”

line 124 - disposal instead of removal

Sentence has been removed.

line 121-123 if potassium dichromate is the preferred method of use in the lab or the particular country that it’s absolutely fine; potassium dichromate though is still toxic and its disposal is not only inconvenient or labour intensive it’s also covered by specific regulations and paperwork. Not sure exactly the word “convenient” is the word you are after.
Also, the studies you cite have also shown that stool is find at RT for a few days too until processed

Section has been re-written as follows (line 106-10):

“Ethanol has been validated for use as a preservative in the absence of cold-chain [1-4], but transport in large volumes by air becomes restricted and costly due to its classification as a dangerous good. Potassium dichromate is commonly used in STH field trials to provide long-term preservation of stool at ambient temperatures and does not have the same freight restrictions [3, 5, 6].”

line 129 - perhaps you can review the very latest literature - <https://pubmed.ncbi.nlm.nih.gov/28268038/>

We have reviewed the suggested research, and do not believe it adds to our manuscript since it reports on a different parasite in a non-human host.

line 137-139 I do not understand the rationale of this ..what was wrong with DNA extraction before?

We have added further detail to clarify the aims of our work:

Lines 110-13:

“Once the potassium dichromate is washed off, the stool must undergo DNA extraction within 4 weeks otherwise the STH DNA may degrade due to removal of the preservative [6]. It remains unknown whether washed stool could be stored and processed for DNA extraction beyond this time.”

Lines 115-20:

“Several researchers have shown that bead-beating is essential when extracting helminth DNA from human stool but in the absence of a standardised protocol it remains unclear if some homogenizers are superior to others [1, 2, 7-9]. Furthermore, bead-beating times range from 30 seconds to 3 minutes [2, 6, 10] and whilst these may seem like minor details, the process of DNA extraction underpins the success of STH detection.”

And lines 129-33:

“Whilst evaluating this staged approach, we sought to fill these technical gaps with the overall aim of facilitating a more effective process. Specifically, we: (i) investigated whether washed stool could be stored below -30°C to allow for DNA re-extraction; (ii) optimised the cycle time for homogenisation of stool during DNA extraction; and (iii) incorporated simple and qualitative analysis of extracted DNA.”

line 140 - you mean qualitative analysis of extracted DNA?

Sentence amended to reflect reviewer’s suggestion, as follows (line 133):

“(iii) incorporated simple and qualitative analysis of extracted DNA.”

line 164- 166 how do you ensure that hookworm DNA released from degraded eggs is not washed away with multiple rinses to remove the potassium dichromate

During washing of the stool we undertook precautionary measures to prevent loss of DNA by performing centrifugation at a slow speed as stated in the Methods (line 156, page 7):

“Briefly, a wooden spatula was used to mix the preserved stool and up to 7.5 ml was poured into a 50 ml Falcon tube and centrifuged at 700 x*g* for 3 minutes, to avoid lysis of worm eggs.”

We have added a limitation to the Discussion as follows (lines 426-28):

“The requirement for multiple rinses to remove the potassium dichromate may have also resulted in loss of free helminth DNA from degraded eggs, however most would be kept intact within eggs or larvae until DNA extraction was performed.”

line 168 - I do not understand what qualification means next to the DNA - please rephrase throughout manuscript

We have made the following changes for clarification as follows:

Line 28: replaced ‘quality control’ with ‘DNA extraction control’

Line 133: replaced ‘incorporate a simple and cost-effective DNA qualification step’ with ‘incorporated simple qualitative analysis of extracted DNA.’

Line 164: replaced ‘DNA extraction and qualification’ with ‘DNA extraction and pre-testing checkpoint’

Line 170: replaced ‘DNA qualification’ with ‘qPCR for the mitochondrial 16S rRNA gene’

Line 343: inserted word ‘extracted’

Line 379: we used the mt qPCR as a proxy for assessing the extracted DNA prior to multiplexing.

Line 684: Amended S1 Fig title to “qPCR of 40 DNA extracts for the vertebrate mitochondrial 16S rRNA gene qPCR”

line 176 - what was the concentration of the probe

The qPCR to detect the mitochondrial 16S ribosomal RNA gene was SYBR® green-based chemistry, and as such did not utilise a probe. This was intentional to keep costs at a minimum.

Line 182 - change Ct throughout - it’s Cqs - see MIQE guidelines bY Bustin, 2009

‘Cycle threshold’ changed to quantification cycle (C_q_) throughout manuscript, tables and figures.

line 207 - I am not sure I understand the meaning - rephrase?

We have rephrased the sentence for clarity as follows (lines 226-29):

“Due to the inherent limitations of specification with KK, when we compared KK and qPCR results we considered any positive qPCR result for *Ancylostoma duodenale,* *Ancylostoma ceylanicum* or *Necator americanus* to be hookworm positive, and *S. stercoralis* results were excluded from the comparison.”

line 240 - it is impossible to compare Ct values across runs, let alone among different assays/worms - It is unrealisable to do interpolate comparison using Ct values - Ct values is meaningless.

We disagree. Comparison of C_q_ within the same worm species is widely used for optimisation of protocols [1-3]. We did not compare C_q_s for *A. lumbricoides* to C_q_s for *S. stercoralis*, nor did we attempt to quantify original template in stool. We performed relative comparisons between C_q_s within the same species to ascertain DNA template per qPCR. Thus, we believe that our interpretation of C_q_s is valid and reasonable.

line 263 - if standard curves were used then why you did not do quantification of your target and you keep using Ct values? Ct values are only somewhat meaningful in gene expression studies and should never be used for Dx

Our standard curves were generated using synthetic gene sequences (gBlocks®, IDT) and as such we could not convert the weight of the gBlocks® to actual worm eggs. The standard curves served to assess the dynamics of the qPCR reactions for both single and multiplex reactions.

line 307 - that is actually useful - again, Ct values should not be used for comparison

Please see our response to your concern at the top of this page. It would have been our preference to use worm eggs for each species throughout our study to validate our methods, and express our data in eggs per gram of stool, however, we did not have access to them. We highlight this challenge in our Discussion section (lines 432-35):

“Another challenge we faced, which is shared by others in the field, is the difficulty in obtaining worm eggs to use as controls [11, 12]. In order to implement a universal qPCR to diagnose these STH, there is a need for a global bank of eggs, larvae and DNA made available, as there is for other parasites.”

Perhaps highlight that you tried Trichuris - most significant finding in my view so far
We have emphasised this by changing the section headings (lines 211 and 316) to:

**“Detection of *Trichuris* in spiked-stool”**

In addition, we have moved the data relevant to this finding from the supplementary section to the main manuscript (now figure 5, line 333, page 17).

line 318 - not sure how this workflow is better/adds values to the already established workflows

This has been reinforced in the concluding remarks of the manuscript:

Lines 440-48:

“This is the only study to present a qPCR workflow to detect this unique combination of six important STHs affecting 1.4 billion humans worldwide [11]. We utilised two multiplex qPCR reactions to detect *Ancylostoma duodenale* and *ceylanicum, Necator americanus*, *Ascaris lumbricoides*, *Trichuris trichiura* and *Strongyloides stercoralis*. In the absence of a ‘gold standard’ for diagnosis of STH infections by qPCR, this work could be very useful towards development of a standard diagnostic test. The methods can be applied for samples collected from remote settings and processed in low resourced molecular laboratories where infections with these parasites thrive.”

line 330 - paragraph - I do not think many labs are using potassium dichromate and that they would be worried to go back and re wash the stool to be reextracted - In fact the majority of the large studies use coolers or ethanol for storing samples

Potassium dichromate is still used in remote communities that do not have access to electricity and cold storage. It can be easily prepared and transported by air and provides long term preservation of stool in humid and warm climates. The procedures detailed in this manuscript extend beyond washing off the potassium dichromate; so even if the use of this perseverative becomes irrelevant, the DNA extraction (with comparisons of bead-beating machines) and qPCR protocols can still be applied to detect up to six STH from any DNA sample.

line 347 - please cite labs that use Fast prep 5g with longer cycles than 40 seconds

We have inserted references for publications that use the FastPrep-24™ 5G for longer than 40 seconds, line 369.

## Reviewer #2

(No Response)

Reviewer #3

The objectives are clearly stated, the population has been described and there are no ethical concerns. However, there is little statistical rigour as a very small sample size was used (n=40) and for the optimisation steps replicates were not used (single data points only). Therefore this has impacted the significance of the findings as they cannot be proven to be significant. Further some of the methodologies used don't appear to be suitable to answer the desired question/objective.

We agree that multiple replicates are necessary to produce robust data. The optimisation steps were performed with replicates whenever possible. We have highlighted this further by adding number of replicates to the results sections and to all figure legends. In addition, we have performed statistical analyses using data in figures 2, 3 and 5 (lines 242, 257 and 326, respectively) to support the significance of these findings.

We have added further data to Table 3 (line 308, page 16) regarding the number of hookworms detected, and corrected a data entry error for the ‘Any parasite’ row. Statistics have been amended accordingly.

Regarding this, vertebrate (host) 16s genes were used to determine quality of DNA extraction, however no evidence was given to suggest that the amount of 16s markers if a known and reliable quantity. The authors should have used the EHV-4 as an internal control at the extraction phase as this would have been a known a predictable amount that would get added to each sample and therefore the Ct values of this marker could then be used to determine successful extraction and possible inhibitory effects. It is not possible to determine if a poor DNA extraction of inhibitors leads to poor Ct values using a genetic marker as they both compound each other. Inhibitory effects can be tested by using a dilution series (this dilutes the inhibitor) meaning that if an inhibitor is present the more dilute the sample the better the efficiency (even if the Ct decreases).

The mitochondrial qPCR checkpoint was used to assess the success of DNA extraction before proceeding to stage 3 (the STH qPCRs), and has been used similarly before [13]. This prevents wastage of more expensive reagents later on (probe chemistry, primers and probes) if the DNA extract is poor (C_q_ >25).

Combining the index tests (qPCR) with the reference test (KK) to make the pseudo-gold standard is a little dubious as it prevents a fair assessment of the sensitivity and specificity due to the pseudo-gold standard being weighted in favour of the index test due to the inclusion of its positive results. No calculation of the specificity against field samples was performed.

We agree that the pseudo-gold standard has its limitations and we have added a sentence in our discussion to reflect this (line 428-31):

“Another limitation is the use of the pseudo gold standard for comparison of qPCR and KK data, as the pseudo-gold standard is weighted in favour of the qPCR (because of inclusion of its positive results); yet this type of analyses has been used in the absence of a true gold standard [14-17].”

# Results

## Reviewer #1

(No Response)

## Reviewer #2

Material&Methods, Results and discussion are not logically structured, a lot the methods is described in the results and the results contain a lot of discussion.

We appreciate the concern raised by Reviewer 2. This manuscript describes methods and optimisation experiments. It can be difficult to separate the methods from the results and discussion, nevertheless, we have attempted to improve the flow of the paper by making the following changes:

- New headings in the Methods have been made to reflect the same headings in Results.
- We have amended figure 1 to emphasize the 3-staged approach of our workflow.

## Reviewer #3

The analysis presented does match the analysis plan and the results are clearly presented in the figures, however, it would be advisable to replace table 2 with a simpler 2x2 table with sensitivity and specificity values included (plus CI).

We have included the essential information that is required for publication of qPCR experiments as recommended by the MIQE guidelines [18]. We have removed threshold values as these are not considered essential data. Specificity testing was not performed in this study. Validation of the qPCR workflow to microscopy was performed and presented in the Results section, Table 3 (line 308, page 16).

Conclusions

## Reviewer #1

I found interesting the small experiment with Trichuris because it has been indeed problematic. Not Ascaris though.

Thank you for your interest in the Trichuris experiment. As mentioned on page 6 of this document, we have reinforced the relevant findings.

It is not an optimised workflow of any sorts; it is a standard procedure for getting DNA out of stool stored preserved in a pretty toxic chemical. I can't think of many labs in the field using potassium dichromate so I am not sure it's of interest in the greater field of STHs.

Potassium dichromate is still used in remote communities that do not have access to electricity and cold storage. It can be easily prepared and transported by air and provides long term preservation of stool in humid and warm climates. The procedures detailed in this manuscript extend beyond washing off the potassium dichromate; so even if the use of this perseverative becomes irrelevant, the DNA extraction (with comparisons of bead-beating machines) and qPCR protocols can be applied to detect up to six STH from any DNA sample.

Perhaps you can highlight the strengths of the manuscript differently. SYBR green is indeed cheaper than Taqman chemistry - but running 2 multiplex experiments i am not sure is more time saving than running one multiplex - the money you save in reagents you put in labour.

As mentioned on page 8, the mitochondrial qPCR checkpoint in stage 2 is used to assess success of DNA extraction before proceeding to the next qPCR multiplex stage. This prevents wastage of more expensive multiplex reagents if the DNA extract is poor (C_q_ >25). It is unlikely that molecular laboratories without automation would have a qPCR machine with capabilities to detect more than 4 targets per reaction. Since we had 6 targets with an additional control, there was no option but to have 2 multiplex reactions.

The MS contains lots of small experiments without an ultimate purpose or highlighting its strengths.

This manuscript is fundamentally a methods paper documenting a workflow made up of small experiments with one ultimate aim- to detect six important STH in humans by qPCR. We prioritised efficiency and costing so that these methods can be incorporated in a non-automated molecular lab to test stools collected from remote settings with limited preservation options.

Similar research in the area (doi):
How is yours different?

We believe our results have not been addressed in previous literature, including the papers referenced. None of these publications compare two popular homogenisers or investigate storage of washed stool beyond 4 weeks at 4℃, nor do they present a single qPCR multiplex for *A. lumbricoides*, *T. trichiura* and *S. stercoralis.* We have provided commentary on all the DOIs listed by the reviewer below, indicating points of difference for our paper.

10.1016/j.mimet.2007.11.007

J Microbiol Methods 2008 Feb;72(2):124-32.

Fecal collection, ambient preservation, and DNA extraction for PCR amplification of bacterial and human markers from human feces.

[Jordan M Nechvatal](https://pubmed.ncbi.nlm.nih.gov/?sort=date&term=Nechvatal+JM&cauthor_id=18162191) [^1^](https://pubmed.ncbi.nlm.nih.gov/18162191/#affiliation-1), [Jeffrey L Ram](https://pubmed.ncbi.nlm.nih.gov/?sort=date&term=Ram+JL&cauthor_id=18162191), [Marc D Basson](https://pubmed.ncbi.nlm.nih.gov/?sort=date&term=Basson+MD&cauthor_id=18162191), [Phanramphoei Namprachan](https://pubmed.ncbi.nlm.nih.gov/?sort=date&term=Namprachan+P&cauthor_id=18162191), [Stephanie R Niec](https://pubmed.ncbi.nlm.nih.gov/?sort=date&term=Niec+SR&cauthor_id=18162191), [Kawsar Z Badsha](https://pubmed.ncbi.nlm.nih.gov/?sort=date&term=Badsha+KZ&cauthor_id=18162191), [Larry H Matherly](https://pubmed.ncbi.nlm.nih.gov/?sort=date&term=Matherly+LH&cauthor_id=18162191), [Adhip P N Majumdar](https://pubmed.ncbi.nlm.nih.gov/?sort=date&term=Majumdar+AP&cauthor_id=18162191), [Ikuko Kato](https://pubmed.ncbi.nlm.nih.gov/?sort=date&term=Kato+I&cauthor_id=18162191)

“Purification and PCR amplification of fecal DNA were compared after preservation of stool samples in RNAlater (R) or Paxgene (P), or after drying over silica gel (S) or on Whatman FTA cards (W). Comparisons were made to samples frozen in liquid nitrogen (N2). DNA purification methods included Whatman (accompanying FTA cards), Mo-Bio Fecal (MB), Qiagen Stool (QS), and others…”

These preservation techniques are not suitable for collection of stool in remote locations with no access to electricity and refrigeration. Furthermore, the preservation reagents used such as RNAlater and Paxgene are far more costly than potassium dichromate. This study is not performed in the context of detection of STH in stools, and the extraction methods are not tested for effective release of parasitic DNA.

10.1017/S0031182017000129

Parasitology; **14 March 2017**

Improved diagnosis of Trichuris trichiura by using a bead-beating procedure on ethanol preserved stool samples prior to DNA isolation and the performance of multiplex real-time PCR for intestinal parasites

[MARIA M. M. KAISAR](https://www.cambridge.org/core/search?filters%5BauthorTerms%5D=MARIA%20M.%20M.%20KAISAR%20&eventCode=SE-AU), [ERIC A. T. BRIENEN](https://www.cambridge.org/core/search?filters%5BauthorTerms%5D=ERIC%20A.%20T.%20BRIENEN%20&eventCode=SE-AU), [YENNY DJUARDI](https://www.cambridge.org/core/search?filters%5BauthorTerms%5D=YENNY%20DJUARDI%20&eventCode=SE-AU), [ERLIYANI SARTONO](https://www.cambridge.org/core/search?filters%5BauthorTerms%5D=ERLIYANI%20SARTONO%20&eventCode=SE-AU), [MARIA YAZDANBAKHSH](https://www.cambridge.org/core/search?filters%5BauthorTerms%5D=MARIA%20YAZDANBAKHSH%20&eventCode=SE-AU), [JACO J. VERWEIJ](https://www.cambridge.org/core/search?filters%5BauthorTerms%5D=JACO%20J.%20VERWEIJ%20&eventCode=SE-AU), [TANIAWATI SUPALI](https://www.cambridge.org/core/search?filters%5BauthorTerms%5D=TANIAWATI%20SUPALI%20&eventCode=SE-AU) and [LISETTE VAN LIESHOUT](https://www.cambridge.org/core/search?filters%5BauthorTerms%5D=LISETTE%20VAN%20LIESHOUT%20&eventCode=SE-AU)

*“*This study aims to improve detection of *T. trichuria* DNA in human stool by evaluating four sample preparation methods….Findings confirmed bead-beating before DNA extraction to be a highly efficient procedure for the detection of T. trichiura DNA in stool.”

This is an important publication for extraction of *T. trichiura* DNA from stool preserved in ethanol (or frozen) and we have referenced this work throughout our manuscript (lines 96, 110, 125, 127-8, 389, 391, 442). Three multiplex qPCRs are used to detect six helminth species and four species of intestinal protozoa. Our manuscript is different in that we used potassium dichromate for preservation which was ideal for our trial location (Fijian islands). We showed that washed stool could be processed for DNA extraction beyond 4 weeks if stored at -30℃. The mitochondrial qPCR checkpoint was used to assess success of DNA extraction before the qPCR multiplex stage to potentially avoid wastage of more expensive multiplex reagents if the DNA extract is poor (C_q_ >25). We chose to focus on six clinically relevant STH and so we configured the STH1 multiplex to detect *A. lumbricoides*, *T. trichiura* and *S. stercoralis*. This combination has never been published and would be useful for diagnosis of these STH, alongside the multiple species of hookworm in our STH2 multiplex [11].

10.1371/journal.pntd.0007778

PLOS NTD

Comparison of four DNA extraction and three preservation protocols for the molecular detection and quantification of soil-transmitted helminths in stool.

Mio Ayana, Piet Cools, Zeleke Mekonnen, Abdissa Biruksew, Daniel Dana, Nour Rashwan, Roger Prichard, Johnny Vlaminck, Jaco J. Verweij, Bruno Levecke

This work further validates the use of potassium dichromate for long-term preservation of stool, and the use of bead-beating to detect *A. lumbricoides*, *T. trichiura,* and *N. americanus*. We present methods for the detection of six clinically relevant STH.

10.1023/A:1020503330767

Conservation Genetics 3: 435–440, 2002.

An evaluation of long-term preservation methods for brown bear (Ursus arctos) faecal DNA samples.

Melanie A. Murphy, Lisette P. Waits, Katherine C. Kendall, Samuel K. Wasser, Jerry A. Higbee & Robert Bogden.

The work above is not relevant to research in diagnosis of STH by qPCR but recommends the use of ethanol as a stool preservative and validates detection of mitochondrial DNA as a proxy to assess yield of extracted DNA.

10.1128/mSystems.00021-16

Applied and Environmental Science

Preservation Methods Differ in Fecal Microbiome Stability, Affecting Suitability for Field Studies.

Se Jin Song, Amnon Amir, Jessica L. Metcalf, Katherine R. Amato, Zhenjiang Zech Xu, Greg Humphrey, Rob Knight

“We recommend the use of 95% ethanol, OMNIgene Gut, or FTA cards for long-term studies and strongly caution against the use of 70% ethanol for any period or unfixed samples over more than a few days.”

Another publication that supports the use of ethanol to preserve stool, however no methods for detection of STH by qPCR.

10.1371/journal.pntd.0006130
PLOS NTD

A comparative analysis of preservation techniques for the optimal molecular detection of hookworm DNA in a human fecal specimen.

Marina Papaiakovou, Nils Pilotte, Ben Baumer, Jessica Grant, Kristjana Asbjornsdottir, Fabian Schaer, Yan Hu, Raffi Aroian, Judd Walson, Steven A. Williams Published January 18, 2018

The authors show that for the detection of *N. americanus* “at 32°C, preservation with FTA cards, potassium dichromate, and a silica bead two-step desiccation process proved most advantageous for minimizing C_q_ value increases, while RNA later, 95% ethanol and Paxgene also demonstrate some protective effect.”

These findings support our use of potassium dichromate as an appropriate stool preservative in tropical climates. No downstream qPCR multiplexes are presented in this study. Furthermore, the internal amplification control is purely a control for qPCR inhibition and not DNA extraction (as it is spiked in as pure plasmid thus requiring no lysis).

## Reviewer #2

Difficult, primary aim was a multiplex PCR for 6 STH and in the field samples Trichuris could not be detected.

We did however detect *Trichuris* in stools spiked with *T. suis* eggs using our extraction method and STH1 qPCR multiplex (line 333, Fig 5, page 17), proving that our multiplex was effective. We have addressed this limitation in the Discussion (lines 399-406).

## Reviewer #3

The conclusions on the whole correspond to the findings however the authors state that they achieved "high sensitivity" for A. lumbricoides and hookworm. Whilst the sensitivity of the qPCR was 75% and 66.7% respectively the low sample number means that the CI for this result will likely be very wide and so somewhat undermines the confidence of making the statement.

We have addressed this limitation in the Discussion previously (lines 411-13):

“Work is currently underway to test a larger sample size. This will allow us to further explore the practicality of this molecular workflow, and to more fully investigate the sensitivity and specificity of our approach compared to microscopy.”

**Editorial and Data Presentation Modifications**

**Reviewer #3**

***The article is well written and clear my only suggestion with regards to presentation would be to change Table 2 to be a more traditional 2x2 table and to include the other diagnostic calculations that are derived from it (sensitivity, specificity, PPV, NPV, PLR, NLR and their corresponding CI.***

As mentioned on page 10:

We have included the essential information that is required for publication of qPCR experiments as recommended by the MIQE guidelines [18]. We have removed threshold values as these are not considered essential data. Specificity testing was not performed in this study, but we compared qPCR to microscopy data using 40 stool samples and have presented this in the Results section, Table 3 (line 308, page 16).

Summary and General Comments

Reviewer #1

Not sure extracting DNA after 4 weeks instead of 3 will make any difference.
Evaluation of storage at various temperatures has occurred before. Potassium dichromate is not an attractive preservative for many reasons.

We showed that DNA can be stored for at least an additional 15 weeks at -30 degrees following washing (line 246, Fig 2, page 12). This allows flexibility in the workflow within low volume laboratories without compromising results.

Similar research in the area (doi):

10.1016/j.mimet.2007.11.007
10.1017/S0031182017000129
10.1371/journal.pntd.0007778
10.1023/A:1020503330767
10.1128/mSystems.00021-16
10.1371/journal.pntd.0006130

Please see comments for each publication on pages 11-14 of our response.

## Reviewer #2

It's is a very nice piece of work, but in the way it is presented it is very difficult to follow. Just describe methods in a logical structured way and follow the same structure presenting the results (without too much discussion) of each part of the methods.

We have improved the flow of the manuscript by using the same headings in the Methods and Results sections. We have amended figure 1, (Workflow of laboratory procedures, line 239, page 12) to emphasize the 3-stage approach that we present in this manuscript.

Abstract

Line 28 Methodology/Principal Findings, what are the principal findings

We have amended the abstract to align with the style for publication in PLOS ONE.

Line 35, is this internal control the same as the quality control in line 32? Later on it turns out to something else, however within the context of the abstract it is not clear.

We have amended the text in the abstract (lines 27 and 31) to clarify the two different controls used: SYBR green qPCR mt DNA extraction control and EHV-4 internal amplification control. Internal viral control has been replaced with internal amplification control throughout the manuscript.

Line 37, is this the same as the high-energy cell disruptor as mentioned in line 31
For consistency, we have replaced “high-energy cell disruptor” with “Mini-Beadbeater-24” (line 33).

Introduction

Line 119/120 why are variances in mixing at collection only a disadvantages for ethanol and not in potassium dichromate?

Variances at mixing and leakage are possible disadvantages for both ethanol and potassium dichromate. We have re-written this section in the introduction (lines 106-10):

“Ethanol has been validated for use as a preservative in the absence of cold chain [1-4], but transport in large volumes by air becomes restricted and costly due to its classification as a dangerous good. Potassium dichromate is commonly used in STH field trials to provide long-term preservation of stool at ambient temperatures and does not have the same freight restrictions [3, 5, 6].”

Line 130, it seems odd that the primary aim detect six clinically important STH affecting humans using qPCR in stool preserved in potassium dichromate was not compared with directly freezing of stool samples and/or preserving the samples in ethanol. Please comment.

The methods we present were chosen for use in our study conducted on Fijian islands where direct freezing of stool (preferred choice) was not an option as highlighted in line 108. Ethanol was not used due to logistical reasons as mentioned above.

Methods
Line 178, were these primers published before (ref?)

Only just recently - reference has been inserted (line 174).

Line 182, how was this threshold (Ct>25) established?

We have added the following details to the methodology (lines 177-78):

“Samples with quantification cycle (C_q_s) >25 (determined using mean+3SD from DNA extracts of 40 stools) were re-extracted using frozen-washed stool, and tested again (S1 Fig).”

Line 184, Ascaris are not the most difficult eggs to crack, why was chosen for Ascaris and not for Trichuris (these are the difficult ones)

Unfortunately, we did not have access to *Trichiura* eggs until the end of our study, when we obtained a very limited amount for our *T. suis* testing. We have added the following (line 195):

“Hookworm and *Trichiura* eggs were not available for comparison.”

Line 201, if I understand correctly the EHV-4 control was not added to the sample before extraction, but used as DNA within the PCR only? Usually an internal control is taken from the beginning, why was chosen to do a separate quality control first (extra costs)

The mitochondrial 16S rRNA qPCR served as a DNA extraction control to prevent wastage of more expensive probe-based reagents (Stage 3: STH qPCRs) of the workflow. The EHV-4 viral DNA was spiked into the qPCR master mix to control for reaction inhibition. We have justified this throughout as follows:

Lines 342-44:

“We incorporated a quality checkpoint to validate extracted DNA using SYBR qPCR targeting a vertebrate mitochondrial gene. If C_q_s were ≤25, DNA underwent final testing in the STH1 and STH2 multiplexes, and if C_q_s were >25, DNA isolation was repeated.”

Lines 348-49:

“Both qPCRs incorporated an internal amplification control using EHV-4 to ensure that negative results were real and not due to reaction inhibition.”

Lines 376-82:

*“*To validate the quality of extracted DNA before continuing onto the multiplex qPCRs, we implemented the first quality control checkpoint in stage 2 of our workflow - amplification of a vertebrate 16S rRNA mitochondrial gene. To rule out the presence of inhibitors within stool and the possibility of potassium dichromate-carryover, we used the mt qPCR as a proxy for assessing the extracted DNA prior to multiplexing. Use of SYBR-based chemistry in a small reaction volume of 10 µl minimised costs and expended only 4 ul of stool DNA (2 ul in duplicate).”

Lines 441-46:

“The EHV-4 internal amplification control in stage 3 provided additional quality assurance of sample preparation in real-time. Each qPCR reaction was spiked with the viral gBlock^®^ (synthetic DNA) and detected on the fourth ROX channel. In our 40 samples, the EHV-4 C_q_ in each well did not deviate by more than 2 C_q_s above the negative control wells, giving us confidence that our systematic approach yielded DNA that could be reliably amplified using robust qPCR reagents manufactured to resist PCR inhibitors.”

Results
How many samples were collected?

For the purposes of this study, 40 field samples were used to validate our methods. We have added this to the methods section (line 139), and it is also mentioned elsewhere (lines 36, 178, 294, 349, 392 and 684).

It would be helpful if the headings of the headings and organization in the material and methods are the same as in the results. E.g. Line 260-261, analytical sensitivity using gblocks are not mentioned in the methods.

Manuscript has been re-structed to improve flow as discussed on page 9. Further information has been added to the manuscript to clarify the use of gBlocks^®^.

Line 205-06:

“These gBlocks® were also used as positive controls for validation of field samples.”

Lines 277-79:

“Both qPCRs were performed in singleplex and multiplex formats using synthetic gBlock® DNA to ensure that sensitivity of gene amplification was not lost due to competition in the multiplex PCR targeting the three parasites (Fig 4).”

Lines 284-287 (Fig 4 legend, pages 13-14)

**Fig 4. The STH1 and STH2 qPCRs performed as both singleplex and multiplex reactions.** Synthetic gBlocks® (IDT) mimicking sequences from each of the six parasites were diluted 10-fold to generate 7-point standard curves. Mean C_q_ values and standard deviations from duplicate wells are shown.”

Line 289 (Table 2 legend, page 14):

“Reactions were performed in a total volume of 25 µl containing 5 µl of synthetic gBlock® DNA (IDT).”

Line 240-241, No difference in the Ct values for Strongyloides between the two disruption methods, indeed that is a result, but the fact that the Ct values for Strongyloides are lower than those for Ascaris is that a result? Did you expect something else? It is just a matter of input.

We have removed the comparison of C_q_s between species.

Line 240-241, Ascaris 5000 EPG results in Ct values 32-33.3, this does not seem very sensitive as usually Ct 35 is around the detection limit of most cyclers.

This data is in line with similar studies [2]. Every cycler produces different dynamic ranges.

Table 2, not clear what the “Threshold”, EPG? Copies, Grams?

In accordance with MIQE guidelines, we have removed ‘threshold’ from Table 2 as it is an unnecessary detail referring to the automated threshold setting for the cycler [18].

The data presented in Table 2 (line 288, page 14) show details of the linear regression analyses used to generate the graphs in figure 4. To clarify this data we have added additional information regarding the template used.

Line 289 (Table 2 legend, page 14):

“Reactions were performed in a total volume of 25 µl containing 5 µl of synthetic gBlock® DNA (IDT).”

Line 285-287 This is not really a proof that mixed infections are picked up if they are present in any ratio, this should be part of a the analytical validation user checkerboard titrations.
We disagree. We have presented mixed titrations in figure 4 of the manuscript showing that up to three STH can be simultaneously detected (STH1: *A. lumbricoides, T. trichiura and S. stercoralis*, and STH2: *A. duodenale*, *A. ceylanicum* and *N. americanus*) down to of 2.4 x 10^-4^ pico grams per reaction.

Line 387, this is not a completely fair comparison, in the DNA extraction 200mg stool is used, as soon as eggs are present for extraction and DNA is released, target copies are present for amplification.

We think it is a fair comparison. Target copies are present for amplification but they are still present in a limited concentration and subject to dilution. The entire 200 mg is not tested in a qPCR reaction. 200 mg is eluted in 100 µl final elution volume, of which 5 µl (5%) is used per qPCR reaction. This equates to 10 mg of original stool.

Reviewer #3

First I wish to commend the authors on are very well written and presented paper, I also wish to mention that the subject they are looking at is an important one. There is an urgent need to address the shortfall in highly sensitive and specific assays for the detection of STH infections in resource poor areas.
However, the study itself is very small, with very few replicates (if any) and only 40 field samples. I appreciate that this may be due to feasibility issues but it is still a limiting factor.

We have acknowledged this as a limitation in our Discussion (page 20):

Line 411-13:

“Work is currently underway to test a larger sample size. This will allow us to further explore the practicality of this molecular workflow, and to more fully investigate the sensitivity and specificity of our approach compared to microscopy.”

The novelty of the assay developed is also limited, there are many examples of multiplex qPCRs for the detection of helminths.

This is the only study to present a qPCR multiplex to simultaneously detect A. lumbricoides, S. stercoralis and T. trichiura. When used with the hookworm multiplex, our workflow can detect six important STHs that represent the ‘diseases of poverty’ as described by Papaiakovou et al [11].

Whilst I believe the work done was to a high standard and it has been very well presented I believe the scope is too small and lacks statistical rigour for publication in PLOS NTD. It also doesn't help that the proposed assay requires each sample to be processed by at least three times, ideally an improved assay would be able to detect all target helminths within one reaction.

We agree that one reaction would be ideal. However, current technology does not exist to allow for 6 STH targets and an internal amplification control to run simultaneously. We utilised the maximum available channels on our cycler per reaction.

This is an expensive approach and the SYBR assay is redundant if the EHV-4 is used as the internal positive control as opposed to the 16s vertebrate qPCR, which itself is likely unreliable for reasons previously mentioned.

As mentioned in responses above (pages 18-19), there are two quality control checkpoints in the workflow with different purposes.

It was also not explained how the CT cut-off of >25 was decided with regards to the results for the 16s qPCR.

We have added the following details to the Methodology (lines 177-79):

“Samples with quantification cycle (C_q_s) >25 (determined using mean+3SD from DNA extracts of 40 stools) were re-extracted using frozen-washed stool, and tested again (S1 Fig).”

Minor comments:
line 139: need to include the following notation "(ii)"

We have corrected as suggested, line 131.

No mention of negative extraction controls

We have added this detail to the Methods (lines 179-80):

“DNA from healthy stools (triplicate) was extracted and served as a negative control for each optimisation experiment.”

Workflow of laboratory procedures, move to methods section

When we moved this to the Methods section it didn’t improve the flow, so it remains at the beginning of the Results section. As this is a methodology manuscript, we consider the 3-staged workflow to be a pivotal result of this study.

line 225: washed stools were stored for up to 4 weeks prior to qPCR or unwashed stools were stored for up to 4 weeks prior to extraction?

We have clarified as follows (line 183-87):

“DNA was extracted from 12 stools following washing and storage at 4°C for up to 4 weeks. The same washed stools were then stored at -30°C for various lengths of time: 1 week (n=6), 5 weeks (n=4) and 15 weeks (n=2); and DNA was extracted again. SYBR green-based qPCR was performed on paired DNA samples in duplicate, to detect the mt 16S rRNA gene and resulting C_q_s were compared.”

stating a method is scalable is a little vague and considering each sample is to undergo 3 seperate qPCRs I would argue that this prevents it from being scalable as one of the biggest costs of qPCR is the mastermix cost, therefore the more assays per sample the more costly.

We were very conscious of costs when designing the methods of this workflow. By performing the DNA pre-testing (mitochondrial qPCR) before the STH qPCRs, we were able to avoid wastage of probe-based reagents on poor DNA extracts (C_q_>25). Furthermore, we maximised the number of targets per STH qPCR reaction given the limitations of the light-cycler. If future studies (using a larger cohort of samples) reveal that most DNA extractions pass the mitochondrial checkpoint, we would consider forgoing this qPCR, and proceed straight to stage 3- the STH qPCRs.

My final comment to the authors is to not be discouraged by this review, although I don't believe it is suitable for publication in PLOS NTD I do believe it has been well written and the work done has been done well. If the article is rejected from PLOS NTD following the other reviewers comments I encourage the authors to re-submit the article to another journal and wish them good luck.

Thank you.

**References**

1.	Ayana M, Cools P, Mekonnen Z, Biruksew A, Dana D, Rashwan N, et al. Comparison of four DNA extraction and three preservation protocols for the molecular detection and quantification of soil-transmitted helminths in stool. PLoS Negl Trop Dis. 2019;13(10):e0007778. Epub 2019/10/29. doi: 10.1371/journal.pntd.0007778. PubMed PMID: 31658264; PubMed Central PMCID: PMCPMC6837582.

2.	Kaisar MMM, Brienen EAT, Djuardi Y, Sartono E, Yazdanbakhsh M, Verweij JJ, et al. Improved diagnosis of Trichuris trichiura by using a bead-beating procedure on ethanol preserved stool samples prior to DNA isolation and the performance of multiplex real-time PCR for intestinal parasites. Parasitology. 2017;144(7):965-74. Epub 2017/03/16. doi: 10.1017/S0031182017000129. PubMed PMID: 28290266; PubMed Central PMCID: PMCPMC5471844.

3.	Papaiakovou M, Pilotte N, Baumer B, Grant J, Asbjornsdottir K, Schaer F, et al. A comparative analysis of preservation techniques for the optimal molecular detection of hookworm DNA in a human fecal specimen. PLoS Negl Trop Dis. 2018;12(1):e0006130. Epub 2018/01/19. doi: 10.1371/journal.pntd.0006130. PubMed PMID: 29346412; PubMed Central PMCID: PMCPMC5773002.

4.	Song SJ, Amir A, Metcalf JL, Amato KR, Xu ZZ, Humphrey G, et al. Preservation Methods Differ in Fecal Microbiome Stability, Affecting Suitability for Field Studies. mSystems. 2016;1(3). Epub 2016/11/09. doi: 10.1128/mSystems.00021-16. PubMed PMID: 27822526; PubMed Central PMCID: PMCPMC5069758.

5.	Stracke K, Clarke N, Awburn CV, Vaz Nery S, Khieu V, Traub RJ, et al. Development and validation of a multiplexed-tandem qPCR tool for diagnostics of human soil-transmitted helminth infections. PLoS Negl Trop Dis. 2019;13(6):e0007363. Epub 2019/06/18. doi: 10.1371/journal.pntd.0007363. PubMed PMID: 31206520.

6.	Llewellyn S, Inpankaew T, Nery SV, Gray DJ, Verweij JJ, Clements AC, et al. Application of a Multiplex Quantitative PCR to Assess Prevalence and Intensity Of Intestinal Parasite Infections in a Controlled Clinical Trial. PLoS Negl Trop Dis. 2016;10(1):e0004380. Epub 2016/01/29. doi: 10.1371/journal.pntd.0004380. PubMed PMID: 26820626; PubMed Central PMCID: PMCPMC4731196.

7.	Cunningham LJ, Odoom J, Pratt D, Boatemaa L, Asante-Ntim N, Attiku K, et al. Expanding molecular diagnostics of helminthiasis: Piloting use of the GPLN platform for surveillance of soil transmitted helminthiasis and schistosomiasis in Ghana. PLoS Negl Trop Dis. 2018;12(1):e0006129. Epub 2018/01/26. doi: 10.1371/journal.pntd.0006129. PubMed PMID: 29370166; PubMed Central PMCID: PMCPMC5784883.

8.	Cunningham LJ, Stothard JR, Osei-Atweneboana M, Armoo S, Verweij JJ, Adams ER. Developing a real-time PCR assay based on multiplex high-resolution melt-curve analysis: a pilot study in detection and discrimination of soil-transmitted helminth and schistosome species. Parasitology. 2018;145(13):1733-8. Epub 2018/08/29. doi: 10.1017/S0031182018001361. PubMed PMID: 30152296.

9.	Inpankaew T, Schar F, Dalsgaard A, Khieu V, Chimnoi W, Chhoun C, et al. High prevalence of Ancylostoma ceylanicum hookworm infections in humans, Cambodia, 2012. Emerg Infect Dis. 2014;20(6):976-82. Epub 2014/05/29. doi: 10.3201/eid2006.131770. PubMed PMID: 24865815; PubMed Central PMCID: PMCPMC4036766.

10.	Andersen LO, Roser D, Nejsum P, Nielsen HV, Stensvold CR. Is supplementary bead beating for DNA extraction from nematode eggs by use of the NucliSENS easyMag protocol necessary? J Clin Microbiol. 2013;51(4):1345-7. Epub 2013/01/18. doi: 10.1128/JCM.03353-12. PubMed PMID: 23325821; PubMed Central PMCID: PMCPMC3666819.

11.	Papaiakovou M, Gasser RB, Littlewood DTJ. Quantitative PCR-Based Diagnosis of Soil-Transmitted Helminth Infections: Faecal or Fickle? Trends Parasitol. 2019;35(7):491-500. Epub 2019/05/28. doi: 10.1016/j.pt.2019.04.006. PubMed PMID: 31126720.

12.	Cools P, Vlaminck J, Verweij JJ, Levecke B. Quantitative PCR in soil-transmitted helminth epidemiology and control programs: Toward a universal standard. PLoS Negl Trop Dis. 2021;15(3):e0009134. Epub 2021/03/05. doi: 10.1371/journal.pntd.0009134. PubMed PMID: 33661910; PubMed Central PMCID: PMCPMC7932098.

13.	Murphy MA, Waits LP, Kendall KC, Wasser SK, Higbee JA, Bogden R. An evaluation of long-term preservation methods for brown bear (Ursus arctos) faecal DNA samples. Conservation Genetics. 2002;3(4):435-40. doi: 10.1023/A:1020503330767.

14.	Knopp S, Salim N, Schindler T, Karagiannis Voules DA, Rothen J, Lweno O, et al. Diagnostic accuracy of Kato-Katz, FLOTAC, Baermann, and PCR methods for the detection of light-intensity hookworm and Strongyloides stercoralis infections in Tanzania. Am J Trop Med Hyg. 2014;90(3):535-45. Epub 2014/01/22. doi: 10.4269/ajtmh.13-0268. PubMed PMID: 24445211; PubMed Central PMCID: PMCPMC3945701.

15.	Glinz D, Silue KD, Knopp S, Lohourignon LK, Yao KP, Steinmann P, et al. Comparing diagnostic accuracy of Kato-Katz, Koga agar plate, ether-concentration, and FLOTAC for Schistosoma mansoni and soil-transmitted helminths. PLoS Negl Trop Dis. 2010;4(7):e754. Epub 2010/07/24. doi: 10.1371/journal.pntd.0000754. PubMed PMID: 20651931; PubMed Central PMCID: PMCPMC2907416.

16.	Knopp S, Rinaldi L, Khamis IS, Stothard JR, Rollinson D, Maurelli MP, et al. A single FLOTAC is more sensitive than triplicate Kato-Katz for the diagnosis of low-intensity soil-transmitted helminth infections. Trans R Soc Trop Med Hyg. 2009;103(4):347-54. Epub 2009/01/27. doi: 10.1016/j.trstmh.2008.11.013. PubMed PMID: 19168197.

17.	Utzinger J, Rinaldi L, Lohourignon LK, Rohner F, Zimmermann MB, Tschannen AB, et al. FLOTAC: a new sensitive technique for the diagnosis of hookworm infections in humans. Trans R Soc Trop Med Hyg. 2008;102(1):84-90. Epub 2007/11/22. doi: 10.1016/j.trstmh.2007.09.009. PubMed PMID: 18028969.

18.	Bustin SA, Benes V, Garson JA, Hellemans J, Huggett J, Kubista M, et al. The MIQE guidelines: minimum information for publication of quantitative real-time PCR experiments. Clin Chem. 2009;55(4):611-22. Epub 2009/02/28. doi: 10.1373/clinchem.2008.112797. PubMed PMID: 19246619.
